# Immunotherapy for hepatocellular carcinoma

**DOI:** 10.1016/j.jhepr.2024.101130

**Published:** 2024-06-09

**Authors:** Alexa Childs, Gloryanne Aidoo-Micah, Mala K. Maini, Tim Meyer

**Affiliations:** 1 Department of Medical Oncology, Royal Free Hospital, London, UK; 2 Division of Infection and Immunity, Institute of Immunity and Transplantation, University College London, London, UK; 3 UCL Cancer Institute, University College London, UK

**Keywords:** hepatocellular carcinoma, immunotherapy, checkpoint inhibitors, tumour microenvironment, biomarkers, adoptive cell therapy

## Abstract

Hepatocellular carcinoma (HCC) is a major global healthcare challenge, with >1 million patients predicted to be affected annually by 2025. In contrast to other cancers, both incidence and mortality rates continue to rise, and HCC is now the third leading cause of cancer-related death worldwide. Immune checkpoint inhibitors (ICIs) have transformed the treatment landscape for advanced HCC, with trials demonstrating a superior overall survival benefit compared to sorafenib in the first-line setting. Combination therapy with either atezolizumab (anti-PD-L1) and bevacizumab (anti-VEGF) or durvalumab (anti-PD-L1) and tremelimumab (anti-CTLA-4) is now recognised as standard of care for advanced HCC. More recently, two phase III studies of ICI-based combination therapy in the early and intermediate disease settings have successfully met their primary end points of improved recurrence- and progression-free survival, respectively. Despite these advances, and in contrast to other tumour types, there remain no validated predictive biomarkers of response to ICIs in HCC. Ongoing research efforts are focused on further characterising the tumour microenvironment in order to select patients most likely to benefit from ICI and identify novel therapeutic targets. Herein, we review the current understanding of the immune landscape in which HCC develops and the evidence for ICI-based therapeutic strategies in HCC. Additionally, we describe the state of biomarker development and novel immunotherapy approaches in HCC which have progressed beyond the pre-clinical stage and into early-phase trials.

**Key points**

• Hepatocellular carcinoma (HCC) arises in a highly tolerogenic organ, on a background of chronic inflammation +/- fibrotic scarring; these influences compound the tolerogenic tumour niche.
• Further research is needed to fully characterise the range of potential therapeutic targets on immune effectors and immune suppressors in early *vs.* advanced HCC.
• Combination therapy with an anti-PD-L1 backbone and either an anti-CTLA-4 immune checkpoint inhibitor (ICI) or anti-VEGF therapy remains standard of care in the first-line setting for advanced HCC.
• Recent phase III trials suggest that anti-PD-L1 in combination with bevacizumab may be effective in intermediate-stage disease when combined with transarterial chemoembolisation, and as adjuvant therapy following resection.
• To date, no predictive biomarkers have been prospectively validated or approved for ICIs in HCC.
• Strategies in development including alternative ICIs, bispecific antibodies and adoptive cell therapies are likely to further re-shape the treatment landscape of HCC.

## Introduction

Hepatocellular carcinoma (HCC) constitutes >90% of primary liver cancers and represents an increasing global health challenge, with over one million individuals predicted to be affected annually by 2025.^1^ The majority of HCC arises in the context of chronic liver disease and geographical incidence varies according to the prevalence of well-described viral and non-viral risk factors. Within Europe there has been a 70% increase in liver cancer-related mortality between 1990 and 2019, partly attributable to the rise in cirrhosis secondary to MASLD (metabolic dysfunction-associated steatotic liver disease).^2^ Late presentation with advanced disease and high recurrence rates or progression following surgical resection or locoregional therapy mean that approximately 50-60% of all patients will ultimately go on to receive systemic therapy for HCC.^3^ The therapeutic landscape of advanced HCC has changed significantly in recent years, with five tyrosine kinase inhibitors (TKIs) and an anti-vascular endothelial growth factor (VEGF) receptor 2 (VEGFR2) monoclonal antibody now approved across both the first- and second-line settings. However, the advent of immunotherapy, and more specifically immune checkpoint inhibitors (ICIs), has transformed the management of advanced HCC and become the backbone of current drug development strategies. First-line treatment with the combination of atezolizumab (anti-programmed death ligand 1 [PD-L1] antibody) and bevacizumab (anti-VEGF antibody) in advanced disease is associated with median survival of approximately 19 months and an objective response rate (ORR) of 30%, thus representing the standard of care to which new regimens are compared.^4^ Research efforts are now focused on improving the survival benefit seen with ICI-based therapy, investigating whether outcomes can also be improved in earlier disease settings and identifying predictive biomarkers of response. In this review, we provide an overview of the immune microenvironment of HCC and the current evidence base for ICI across all clinical stages of HCC. We end by discussing whether predictive biomarkers can help in selecting those patients most likely to benefit from treatment, and novel directions for future immunotherapy-based treatment in HCC.

## The immune landscape of HCC

HCC differs from other cancers in that the majority of cases arise on the background of a diseased organ, with chronic necro-inflammation, often accompanied by fibrosis or cirrhosis, therefore likely to be dominant influences on tumour immunity.^5^ Chronic inflammation is itself indicative of a sub-optimal immune response and a harbinger for tumour initiation, proliferation and progression.^6^^,^^7^ Moreover, the liver has a constitutively tolerogenic immune milieu, mediated by a variety of mechanisms that may be co-opted to further compound the typical immunosuppressive niche found in tumours.^8^^,^^9^ This immunotolerance limits the induction of immunity against innocuous antigens but predisposes the liver to immune evasion by hepatotropic carcinogenic viruses (such as HBV and HCV) and cancer cells alike. In HCC, transformed hepatocytes can therefore avoid immune clearance through complex mechanisms of augmented immune suppression.

**Fig. 1:**
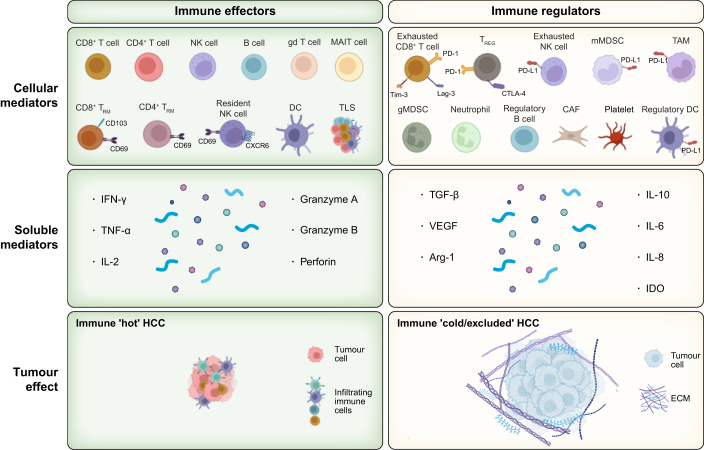
The local HCC immune landscape can be divided into immune effectors and immune regulators. The local HCC immune landscape can be divided into *immune effectors* that can respond to HCC by producing a variety of mediators with anti-tumour potential (*e.g.* tissue-resident T cells, left top and middle panel) and *immune regulators* that suppress and/or exclude these immune effectors through membrane-bound checkpoint inhibitors (*e.g.* PD-L1) and soluble mediators (*e.g.* TGF-b, right top and middle panel). The balance of these opposing activities results in tumour control or growth, respectively. The goal of immunotherapies is to overcome immune exclusion/cold tumours (right bottom panel) and block negative immune regulators to allow influx and function of immune effectors for tumour shrinkage (bottom left panel). Key cellular subsets, soluble mediators and structural elements contributing to these processes are shown. Immune hot tumour – red tumour, immune cold/excluded tumour – blue (bottom panels). CAF, cancer-associated fibroblast; DC, dendritic cell; gMDSC, granulocytic myeloid derived suppressor cell; IL-, interleukin; IDO, idoleamine 2,3 dioxygenase; IFN-y, interferon γ; MAIT cell, mucosal-associated invariant T cell; mMDSC, monocytic myeloid derived suppressor cell; NK cell, natural killer cell; TAM, tumour associated macrophage; TGFβ, transforming growth factor-β; TLS, tertiary lymphoid structure; TNF-α, tumour necrosis factor-α; Treg, regulatory T cell; TRM, tissue resident memory; VEGF, vascular endothelial growth factor.

The immune system plays a dual role in cancer; suppression of tumour growth (by effector cells) and promotion of tumour progression (by immunosuppressive cells) (Fig. 1); thorough assessment of these should help guide prognostic and immunotherapeutic targets.[7], [10] The balance between immune activation and evasion is determined by the opposing action of these cellular components and their soluble mediators, and their relative composition within the tumour microenvironment (TME) has been used to define four subclasses of HCC with distinct clinical outcomes.^11^ There have been significant advances in understanding the full range of HCC immune effectors and inhibitors, particularly by single-cell transcriptomic studies,[12], [13], [14] but these need to be complemented by proteomic and spatial analyses, and improved access to tumour tissue from advanced disease, in addition to early, resectable disease.[15], [16], [17]

### Immune suppressors of the antitumour response

Conventional regulatory subsets including regulatory T cells (Tregs), tumour-associated macrophages (TAMs) and myeloid-derived suppressor cells (MDSCs) are abundant in HCC and cooperate with stromal cells to preserve an immunosuppressive TME. Tregs can promote self-tolerance and suppress excessive immune activation via mechanisms including the production of inhibitory cytokines (*e.g*. interleukin [IL]-10, IL-35 and transforming growth factor-β [TGFβ]), direct cytolysis and metabolic disruption.^18^ Tregs are present in higher numbers within the peripheral blood and TME of HCC compared to normal liver, and higher infiltration corresponds with higher grade tumours, lower disease-free and overall survival (OS).[11], [19], [20]

TAMs cooperate with cancer-associated fibroblasts to form an immune barrier exclusionary to effector cells.^21^ They can additionally recruit Tregs via production of chemokines and further promote immune evasion via the production of immunosuppressive cytokines such as IL-10, expression of the inhibitory checkpoint ligand PD-L1, and downregulation of MHC II/costimulatory molecules required for successful CD8+ T-cell activation.[9], [10], [22] The presence of TAMs correlates with a worse prognosis in HCC, especially if skewed toward an M2 phenotype.[14], [21], [23]

MDSCs are a heterogeneous population of immunosuppressive immature myeloid cells (monocytic or granulocytic), which are abundant in the liver and increased in the peripheral blood of patients with HCC.[24], [25], [26] They promote local Treg differentiation from CD4+ T cells and suppress CD8+ T cell and natural killer (NK) cell activation through mediators such as TGFβ and arginase.^27^ Granulocytic MDSCs overlap with immunosuppressive neutrophils that also accumulate in HCC and represent novel immunotherapy targets.^28^ Other immunosuppressive cell types that have been described in HCC include a subset of B cells that express PD1 and have regulatory functions, CCR4- and CCR6-expressing T helper 17 cells, and tolerogenic dendritic cells expressing high levels of CTLA-4 and PD1.[29], [30], [31], [32]

### Immune effectors of the antitumour response

Infiltrating and tissue-resident CD8+ T cells are the primary effectors of the antitumour immune response, and their presence correlates with improved prognosis and response to treatment.[33], [34], [35] However, CD8+ T cells are often highly dysfunctional within the HCC TME and thus ineffective at tumour clearance. Single-cell studies have shown both a reduction in the number of effector CD8+ cells, as well as a more pronounced “exhaustion” phenotype;^12^ enrichment of exhausted CD8+ T cells at the expense of tissue-resident memory cells is linked to poor survival.^34^

Exhausted CD8+ T cells can express multiple inhibitory receptors such as programmed death 1 (PD-1), cytotoxic T-lymphocyte associated protein 4 (CTLA-4), TIM-3 (also known as HAVCR2), lymphocyte-activating 3 (LAG3) and T cell immunoreceptor with Ig and ITIM domains (TIGIT), which serve as targets for immune checkpoint blockade.[36], [37] Improved understanding of their hierarchical expression by tumour sampling could direct personalised ICI selection in the future. Other key anti-tumour effectors, such as NK cells and γδ T cells, also display an exhausted phenotype, including an exhausted functional and metabolic profile,[37], [38], [39], [40], [41] and represent potential targets for future HCC immunotherapy. A potential contribution of B cells and tertiary lymphoid structures to HCC outcomes is also emerging (Fig. 1).[42], [43]

### Influence of cytokines and other soluble mediators

The HCC TME is enriched in cytokines and other soluble mediators that pleiotropically modulate the composition and function of the immune cells present in HCC. TGFβ, IL-10, IL-6, indoleamine 2,3-dioxygenase and arginase secreted by various cell types in the TME promote immunosuppression. For example, TGFβ produced by tumour cells, macrophages, cancer-associated fibroblasts and Tregs downregulates the antitumour response at different levels, including activation of tolerogenic dendritic cells, M2 polarisation of TAMs, suppression of CD8+ T and NK cells, and generation of Tregs.^44^ High tissue expression of TGFβ is associated with poor prognosis in HCC, and high circulating levels correlate with poorer response to sorafenib and pembrolizumab.[45], [46], [47] Conversely, pro-inflammatory cytokines such as IL-2, interferon (IFN)-y, chemokine C-X-C motif ligand (CXCL)10 and CXCL9 attract effector cells to mount an antitumour response, with IFN-y driving PD-L1, and activation of these pathways has been shown to predict a favourable response to immune checkpoint inhibition.[9], [48], [49] Hence, the interplay of these mediators shapes the immune composition and its ensuing response.

## Impact of aetiology

Diverse immune mechanisms have been implicated in driving liver damage and/or immunosuppression according to the underlying aetiology,[10], [50], [51], [52] but to what extent these are specifically co-opted when tumours arise in these diverse backgrounds is not yet clear. For example, CXCR6+ CD8 T cells implicated in MASH (metabolic dysfunction-associated steatohepatitis)-related immunopathology^53^ and potential resistance to PD-1 blockade^50^ can also mediate bystander damage in HBV.^54^ Similarly, the efficacy of aspirin in HCC prevention^55^ may be underpinned by common or distinct immunological roles for platelets described in HCC arising in HBV^56^ and MASLD.^57^ Direct comparative studies will be needed to determine the contribution of distinct aetiology-driven immune mechanisms to HCC immunopathogenesis and immunotherapy.^37^

## Antigenicity of HCC

Anti-HCC T-cell immunity may be elicited via the abnormal expression of oncofetal and cancer testis antigen genes (AFP, GPC3, MAGE-1 and NYESO1), viral peptide, or tumour-specific neoantigens. CD8+ T cells specific for alpha-fetoprotein (AFP), glypican 3 (GPC3), MAGE-1 (melanoma associated gene 1) and NYESO1 (New York oesophageal squamous cell carcinoma 1) can be detected in the blood and tumours of patients with HCC, and positively correlate with patient survival.[33], [58] In HBV/HCV-associated tumours, neoantigens can be generated by virally encoded open reading frames.[59], [60] Alternatively, genomic mutations may produce tumour-specific neoantigens that can induce naturally occurring anti-tumour T-cell responses. In HCC, circulating CD8 T cells targeting neoantigens are only detected in ∼15% of patients,[33], [59] stimulating interest in developing gene-modified cell therapies directed at HCC neoantigens.

## Immune checkpoint inhibition in HCC

### Advanced-stage HCC

#### Single agent

In both the first- and second-line setting, single-agent immune checkpoint inhibition focuses predominantly on the therapeutic targeting of PD-(L)1 to restore effector CD8+ T-cell function. Initial phase II data for pembrolizumab and nivolumab following first-line sorafenib demonstrated encouraging response rates of 18% and 14% (per RECIST v1.1 criteria) with a prolonged median duration of response (DOR) of 21 and 39.7 months, respectively.[61], [62], [63], [64] The subsequent phase III studies of nivolumab (CheckMate 459) and pembrolizumab (KEYNOTE-240) confirmed the activity and safety of these drugs in the first- and second-line setting, respectively (Table 1), but failed to demonstrate an OS benefit according to pre-defined statistical thresholds when compared to sorafenib or placebo.[65], [66] In contrast, the phase III KEYNOTE-394 study, conducted in an Asian population, demonstrated a statistically significant improvement in both median OS (14.6 *vs.* 13 months; hazard ratio [HR] 0.79; 95% CI 0.63-0.99; *p* = 0.018) and ORR (12.7 *vs.* 1.3%) for pembrolizumab *vs*. placebo in the second line.^67^ A subsequent meta-analysis showed consistent outcomes between the two KEYNOTE studies [41], and it is likely that the statistical design of KEYNOTE 240, including the use of dual primary endpoints of progression-free survival (PFS) and OS and two interim analyses, contributed to its failure to meet the pre-defined criteria for positivity.^68^ Further evidence for the efficacy and safety of single-agent ICI comes from the phase III HIMALAYA trial, in which OS with durvalumab monotherapy was shown to be non-inferior to sorafenib (HR 0.86, 95% CI 0.73-1.03, non-inferiority margin, 1.08) with an improved toxicity profile.^69^ Similarly, the RATIONALE-301 study demonstrated the non-inferiority of tislelizumab with sorafenib in the first-line setting (HR 0.85, 95% CI 0.71–1.02).^70^ This study, in keeping with other ICI studies, demonstrated a higher ORR rate (14.3 *vs.* 5.4%) and median DOR for the immunotherapy arm (36.1 months, 95% CI 18.8 to not evaluable, *vs.* 11 months, 95% CI 6.2-14.7).

**Table 1:** Phase III trials evaluating ICI across HCC stages.

Trial (Ref)	No patients	mRFS (months)	HR RFS	ORR%	mDOR (months)	mPFS (months)	HR PFS	mOS (months)	HR OS	TRAE leading to discontinuation % (% discontinuing ICI alone)
**Adjuvant**										
IMbrave 050^79^										
Atezolizumab/bevacizumab	334	NE	0.72	NE	NE	NE	NE	NE	NE	9
Surveillance	334	NE								
**Intermediate stage**										
EMERALD-1^89^										
TACE + durvalumab + bevacizumab	204			43.6	NE	15	0.77	NE	NE	8.4
TACE + durvalumab	207			41		10	0.94			4.3
TACE	205			29.6		8.2				3.5
**Advanced disease**										
IMbrave150^4^										
Atezolizumab/bevacizumab	336			30	NE	6.9	0.65	19.2	0.66	22 (10)
Sorafenib	165			11	NE	4.3		13.4		12
ORIENT-32^72^										
Sintilimab/bevacizumab biosimilar	380			21	NE	4.6	0.56	NR	0.57	14
Sorafenib	191			4	9.8	2.8		10.5		6
HIMALAYA^69^										
Tremelimumab/durvalumab	393			21.1	22.3	5.4	0.9	16.4	0.78	14
Durvalumab	389			17	16.8	3.8	1.02	16.6	0.86	8
Sorafenib	389			5	18.4	5.6		13.7		17
COSMIC-312^73^										
Atezolizumab/cabozantanib	432			11	12.4	6.8	0.63	15.4	0.9	14 (6)
Sorafenib	217			4	8.4	4.2		15.5		8
LEAP-002^74^										
Pembrolizumab/lenvatinib	395			26	16.6	8.2	0.87	21.2	0.84	18 (6)
Lenvatinib	399			17	10.4	8		19		11(5)
RATIONALE -301^70^										
Tislelizumab	342			14.3	36.1	2.1	1.11	15.9	0.85	10.9
Sorafenib	332			5.4	11	3.4		14.1		18.5
CheckMate 459^65^										
Nivolumab	371			15	23.3	3.7	0.93	16.4	0.85	7
Sorafenib	372			7	23.4	3.8		14.7		11
CARES-310^75^										
Camrelizumab/rivoceranib	272			25	14.8	5.6	0.52	22.1	0.62	24 (4)
Sorafenib	271			6	9.2	3.7		15.2		4

(m)DOR, (median) duration of response; HCC, hepatocellular carcinoma; HR, hazard ratio; ICI, immune checkpoint inhibitor; NE, not evaluable; NR, not reached; ORR, objective response rate; (m)OS, (median) overall survival; (m)PFS, (median) progression-free survival; (m)RFS, (median) recurrence-free survival; TACE, transarterial chemoembolisation; TRAE, treatment-related adverse event.

#### Combination therapy

Whilst ICI monotherapy in advanced HCC has shown encouraging response rates, it has failed to consistently translate into significant improvements in OS. To address this, efforts have focused on developing novel combinations consisting of an anti-PD-(L)1 backbone and (a) monoclonal antibody/multikinase inhibitors with activity against VEGF(R) or (b) additional immune checkpoint inhibition (Table 1). There is a good rationale for combining VEGF/PD-(L)1 blockade in HCC, given the pre-existing evidence of anti-angiogenic efficacy in HCC and the role of VEGF(R) in maintaining an immunosuppressive TME. The phase III IMbrave-150 study of atezolizumab (anti-PD-L1) and bevacizumab (anti-VEGF) therapy was the first to demonstrate a survival benefit over sorafenib in the first-line setting and has become the standard of care in advanced HCC.^4^ Updated efficacy data has shown a median OS of 19.2 months (95% CI 17-23.7) in the atezolizumab/bevacizumab arm compared to 13.4 months (95% CI 11.4-16.9; stratified HR 0.66, 95% CI 0.52-0.85, *p* <0.001) in the sorafenib arm.^71^ Furthermore, the ORR was significantly improved with this combination (30% *vs.* 11%, *p <*0.001) with a median DOR of 18.1 months (95% CI 14.6-not evaluable) for atezolizumab/bevacizumab and 14.9 months (95% CI 4.9-17.0) for sorafenib. Consistent with this, the phase III ORIENT-32 study, which compared the combination of sintilimab (PD-L1 inhibitor) and IBI305 (a bevacizumab biosimilar) to sorafenib in a Chinese patient population, demonstrated an OS benefit for combination therapy (HR 0.57, 95% CI 0.43-0.75).^72^ In contrast, phase III studies evaluating PD-(L)1 with TKI therapy have shown variable results to date. The combination of cabozantinib plus atezolizumab failed to show an OS benefit in the first-line setting compared with sorafenib (15.4 months, 96% CI 13.7–17.7, *vs.* 15.5 months, 96% CI 12.1–not estimable) and reported disappointing response rates of 11% for the combination therapy.^73^ LEAP-002 also failed to meet prespecified significance for improved OS when evaluating lenvatinib and pembrolizumab in combination *vs*. lenvatinib alone (21.2 *vs.* 19.0 months, HR 0.84, 95% CI 0.71-1.00, stratified log-rank *p* = 0.023), in part explained by the unexpectedly long survival of patients in the lenvatinib arm.^74^ However, the combination of camrelizumab (anti-PD-1) and rivoceranib in the first-line setting was recently reported to be associated with a median OS of 22.1 months, the longest observed for any systemic therapy in the first-line setting for advanced HCC, and statistically superior to sorafenib (22.1 months, 95% CI 19.1-27.2, *vs.* 15.2 months, 95% CI 13.0-18.5; HR 0.62, 95% CI 0.49-0.80, one-sided *p <*0.0001).^75^ The study was conducted in a predominantly Asian population, with over 70% of patients having HBV-related liver disease and the outcome of global regulatory review is awaited.

Combining different ICIs has also been explored. The first study to provide evidence of the efficacy of targeting both CTLA-4 (ipilimumab) and PD-1 (nivolumab) in advanced HCC was the phase I/II CheckMate 040.^76^ This demonstrated a promising response rate of 32% in arm A (nivolumab 1 mg/kg plus ipilimumab 3 mg/kg once every 3 weeks) in the second-line setting and the phase III CheckMate 9DW has recently reported meeting its primary endpoint of OS benefit compared to sorafenib or lenvatinib in the first-line setting (NCT04039607). Combined blockade of CTLA-4 and PD-L1 in the STRIDE regimen (tremelimumab and durvalumab, respectively) has also shown benefit over sorafenib in the first-line setting.^69^ In the phase III HIMALAYA study, median OS in the STRIDE arm was 16.4 months (95% CI 14.1-19.5) *vs.* 13.7 months (95% CI 12.2-16.1) for sorafenib, with a superior ORR of 20.1% *vs.* 5.1%. Recently, longer term follow-up data has been reported for the HIMALAYA trial and this has confirmed a durable survival benefit of 25.2% at 4 years *vs.*15.1% for sorafenib.^77^

### Early-stage disease

#### Adjuvant setting

Following the success of the atezolizumab/bevacizumab regimen in advanced HCC, interest has extended to the application of ICI in restoring anti-tumour cellular immune function in the adjuvant setting and four global phase III trials investigating this concept opened in parallel.^78^ IMbrave-050 is the first trial to have reported and demonstrated an improved RFS with atezolizumab/bevacizumab therapy, with a hazard ratio of 0.72 (adjusted 95% CI 0.53–0.98, *p* = 0.012) at the first pre-determined interim analysis, amounting to an absolute risk reduction of 12.5% (95% CI 5.6-19.5) at 12 months.^79^ Eligible patients were classified as high risk for recurrence according to criteria incorporating the number and size of tumours, as well as histological criteria such as the presence of microvascular invasion or poorly differentiated tumours. The event to patient ratio for survival was only 7% and further follow-up will be required in order to address the secondary endpoint of OS. Of note, 61% of the surveillance group who met the RFS event had already crossed over to atezolizumab and bevacizumab at the time of publication. Recently, in an open label phase II trial conducted in six centres in China, sintilimab (anti-PD-1) was also shown to prolong RFS in patients with microvascular invasion when compared to active surveillance after hepatic resection (median RFS, 27.7 *vs.* 15.5 months; hazard ratio 0.534, 95% CI 0.360–0.792; *p* = 0.002).^80^ Notably, adjuvant therapy was given for 6 months compared to 12 months in IMbrave-050. The shorter duration of therapy could offer financial and quality of life benefits to patients and extend therapy to those with contraindications to bevacizumab. However, in both studies the majority or entirety of patients were Asian and most had hepatitis B-related liver disease. There are outstanding questions as to how this result can be applied to a Western population. The results reported to date demonstrate an early efficacy signal for ICIs as adjuvant therapy, but further follow-up and additional trial readouts (NCT03383458, NCT03867084 and NCT03847428) will reveal the extent and durability of the benefit.

#### Neoadjuvant and perioperative setting

Neoadjuvant or perioperative immunotherapy strategies are particularly attractive in HCC, where up to 70% of patients with early-stage disease amenable to surgical resection recur within 5 years and adjuvant sorafenib therapy has failed to show any benefit.[81], [82] There is a biological rationale for immunotherapy in this setting, where increased exposure to tumour-specific neoantigens whilst the disease remains *in situ* may enhance development of anti-tumour immunity. Critically, preoperative therapy also allows for an assessment of drug sensitivity, which may inform the selection of post-operative therapy. Several early-phase trials predominantly targeting patients with upfront resectable disease have been reported to date, with major pathological response rates varying between 17.6% and 33%.[13], [83], [84] A meta-analysis of nine studies (including 193 patients) demonstrated a median major pathological response rate of 27.3% with no single ICI identified as superior in subgroup analysis.^85^ In a phase II study of neoadjuvant nivolumab or ipilimumab-nivolumab, 6 of the 20 patients who underwent resection had a major pathological response, defined as ≥70% necrosis, and importantly none had recurred at 26.8 months of follow-up.^83^ As predicted from data in the advanced setting, the rate of Grade 3/4 immunotherapy-related adverse events was significantly higher in the combination arm (6 [43%] of 14 patients) than in the nivolumab alone arm (3 [23%] of 13; difference 20%, 95% CI −14.7% to 38.7, *p* = 0.69); however, no patients had surgery delayed due to this. Across early phase trials, the incidence of grade ≥3 treatment-related adverse events has varied from 10-30%, with a low surgical delay rate of 1.7% (95% CI 0–4.1%).^85^ However, it is important to note that, in several studies, a significant proportion of patients did not proceed to surgery due to disease progression or other factors, thus the reported pathological response rate is in the per protocol rather than the intention to treat population. There are several ongoing early phase trials investigating ICIs for resectable or borderline resectable HCC. Beyond PD-1 and CTLA-4 blockade, combination strategies incorporating antibodies or TKIs targeting VEGFRs are also being explored. An example of this is the multi-centre PRIMER-1 study, where participants are randomised to 6 weeks of neoadjuvant therapy consisting of pembrolizumab, lenvatinib or pembrolizumab/lenvatinib, followed by a year of adjuvant pembolizumab post-operatively (NCT05185739). These studies will further define the role of immunotherapy in the neoadjuvant or perioperative setting.

### Intermediate-stage disease

Locoregional therapies remain the mainstay of treatment for those with intermediate-stage HCC, or early disease not suitable for surgery or ablation. In view of the prolonged OS benefit seen in the advanced setting, combination therapies targeting both PD(L)1 and VEGFR are now also being investigated in multinodular intermediate-stage disease, with locoregional therapies as the control arm (NCT04803994, NCT04777852). There is also a good biological rationale for combining immunotherapy with locoregional treatment, as embolisation can induce tumour necrosis and enhance tumour antigen presentation.[86], [87], [88] There are several ongoing studies investigating whether clinical outcomes with locoregional therapy can be improved upon by combining with immunotherapy, either as a monotherapy (NCT04268888, NCT04340193) or in combination with anti-VEGF(R)-directed therapy including bevacizumab, lenvatinib and regorafenib (NCT04712643, NCT04340193, NCT04246177). To date, the only study to have reported is EMERALD-1, a global double-blind, randomised, placebo-controlled phase III trial of durvalumab plus transarterial chemoembolisation (TACE) concurrently, followed by durvalumab with or without bevacizumab *vs.* TACE plus placebo(s) in 616 patients with unresectable HCC eligible for embolisation. The study met its primary endpoint, with a significant PFS benefit for TACE plus durvalumab and bevacizumab *vs.* the TACE control (median PFS 15.0 *vs.* 8.2 months; HR 0.77, 95% CI 0.61–0.98, *p* = 0.032).^89^ The full results are awaited and further follow-up will be required to address the secondary endpoint of OS; however, these initial results may indicate a new role for immunotherapy in intermediate-stage disease, whilst also raising questions about the implications for first-line treatment options in the advanced-stage setting.

## Biomarkers

Despite improved clinical outcomes in advanced HCC following the introduction of ICIs, only 30% of patients achieve an objective response and the majority progress. Consequently, there have been intensive efforts to define predictive biomarkers that could inform clinical decision making, reduce unnecessary toxicity and improve overall cost benefit at a population level. For this purpose, it is important to distinguish biomarkers that are merely prognostic, while the methodology for validation of predictive markers has been clearly defined.^90^ Similarly, those biomarkers which are merely associated with response, such as fall in AFP, are less valuable since they can only be measured after a treatment decision has been made. To date, the only predictive biomarker that has been validated in a prospective randomised trial is baseline AFP for the use of ramucirumab.^91^ However, a range of potential biomarkers have been evaluated in retrospective series and as exploratory endpoints in the context of prospective clinical trials.

### Clinical factors

Subgroup analysis of IMbrave-150 trial suggested that patients with non-virally associated HCC did not have the same survival benefit with ICIs as those with HBV/HCV-associated HCC.^4^ Additionally, studies using preclinical models of MASH-induced HCC showed lack of response to anti-PD-1 therapy and, when used prophylactically, led to an increased incidence of HCC associated with an increase in hepatic CD8^+^PD-1^+^CXCR6^+^ T cells.^50^ However, data from numerous trials including IMbrave-150 demonstrate a similar radiological response between viral and non-viral HCC.[4], [61], [92] Moreover, a recent meta-analysis of eight randomised phase III trials confirmed survival benefit for patients treated with ICI-based therapy compared with TKI controls. Based on current data there is insufficient evidence that background liver disease aetiology can be used to predict response to ICIs.^93^

The CRAFITY score has been proposed as a predictive biomarker for responses to ICI treatment in HCC. Using baseline AFP and C-reactive protein, three categories are defined which correlated with survival in patients with HCC treated with anti-PD-(L)1 therapy in both a training and validation cohort.^94^ However, the score was similarly correlated with survival in a sorafenib-treated cohort suggesting that it is generally prognostic. There was some association with radiological response rate, which ranged from 29% in CRAFITY-low to 17% in CRAFITY-high, but this is not sufficient basis on which to make a treatment decision and the score requires prospective validation.

### Tumour mutational burden and PD-L1 expression

Tumour mutational burden (TMB) quantifies the number of mutations per megabase (Mb) in the tumour genome, and those with a high TMB (TMB-H) tend to have more immunogenic neoantigens and greater sensitivity to anti-PD-(L)1 therapy. In 2020, based on a single arm trial in lung cancer, the FDA approved pembrolizumab for tumours with TMB ≥10 mutations/Mb using the FoundationOne CDx assay. HCC tends to be on the lower end of the spectrum with values ranging from 0.42 to 65.6 Mut/Mb and medians ranging from 2.56 to 5 Mut/Mb.^95^ Analysis of the atezolizumab plus bevacizumab-treated patients in IMbrave 150 showed no relationship between TMB and response.^35^ Similarly, in CheckMate 459, there was no significant difference in OS between those patients with high or low TMB treated with nivolumab. Only 3% had microsatellite instability-high tumours and none of these 12 patients showed a response in either treatment arm.^96^

Tumour cell PD-L1 expression by immunohistochemistry is an established predictive biomarker for PD-(L)1 treatment of non-small cell lung cancer^97^ but does not appear to consistently correlate with response in many other tumours including HCC. In the single-arm CheckMate 040 trial, ORR and OS were higher in patients with tumour PD-L1 expression ≥1%, particularly in the sorafenib-experienced group [110] but in randomised trials, including CheckMate 459, IMbrave 150 and HIMALAYA,[71], [77], [96] there was no additional survival benefit for those with tumour PD-L1 expression ≥1%. A more extensive analysis of IMbrave 150 showed no difference in response based on immune cell or tumour cell PD-L1 expression unless expression was ≥10%.^35^ But high expression was only recorded in 14 patients treated with atezolizumab and bevacizumab. Overall, the current data do not support the use of PD-L1 expression as a predictive biomarker.

### Mutations and gene signatures

Mutations in *CTNNB1* resulting in activation of the Wnt/β-catenin pathway are present in around one-third of patients with HCC and are associated with the immune exclusion sub-class.^98^ Initial pre-clinical and patient cohort studies suggested that alterations in Wnt/β-catenin signalling were associated with resistance to ICIs,[99], [100] but subsequent randomised trials have failed to confirm these observations. In both the Checkmate 459 and IMbrave 150 trials, no significant difference in survival was identified in the ICI-containing arms based on *CTNNB1* mutations or Wnt/β-catenin pathway activity.[4], [96] Interestingly, both studies demonstrated that patients treated with sorafenib had improved outcomes in the presence of *CTNNB1* mutations.

Many inflammatory gene signatures have been reported and their association with response and survival has been evaluated in exploratory analyses of prospective trials. In a *post hoc* analysis of 37 patients from the CheckMate 040 trial, several signatures were associated with both response and OS.^49^ The so called atezolizumab plus bevacizumab response signature (ABRS) was derived from the top 10 genes obtained from differentially expressed gene analysis and curated gene signatures using data from the GO30140 study.^35^ The ABRS and inflammatory signature genes, including *CD274* and an effector T cell signature (CXCL9, PRF1 and GZMB), were higher in those achieving a complete/partial response in the IMbrave150 atezolizumab plus bevacizumab-treated group. PFS and OS was also improved in multivariate analysis. Artificial intelligence has been used to impute the presence of the ABRS in histological specimens and this may provide a cheaper and clinically applicable method to select patients in the future.^101^ More recently, an 11-gene signature (IFNAP) defined by upregulation of IFN-γ signalling and MHC II-related antigen presentation was derived from a cohort of patients treated with anti-PD-1 monotherapy.^102^ This signature appears to be associated with outcome in patients treated with anti-PD-1 therapy in the front-line. However, all these studies should be considered as hypothesis generating and require prospective validation in order to qualify them as clinically valuable predictive biomarkers that can be used for clinical decision making.

### The role of biopsy

Tissue-based biomarker research in HCC has been severely limited in the past by reliance on radiological diagnostic criteria. However, the limitations of non-invasive diagnostic criteria in the setting of advanced disease have been clearly demonstrated^103^ and diagnostic tissue biopsy is increasingly routine. It is clearly important that routinely collected tissue is associated with consent for research and linked to well annotated clinical data. Whilst circulating tumour DNA will become an important resource in the future, detailed interrogation of the TME will remain dependent on tissue-based analysis.

## New immunotherapy approaches in clinical trials

The approval of immunotherapy-based therapies in HCC has significantly altered the prognosis for patients with advanced HCC and become the new benchmark for drug development strategies. Current research efforts focus on expanding existing combination therapies and developing novel immunotherapy strategies beyond ICI. Whilst many new immunotherapeutic targets are being explored in pre-clinical studies, herein we will briefly review those that have progressed to clinical trials.

### Novel ICIs

Beyond CTLA-4 and PD-(L)1 blockade, there is interest in targeting alternative immune-checkpoints in HCC to build on the success of combination therapy and overcome resistance mechanisms to ICI-based regimens. The combination of TIM-3 and PD-1 blockade with cobolimab and dostarlimab is currently under evaluation in a single-arm phase II study of treatment-naive patients with advanced HCC.^104^ Interim results have shown encouraging signs of efficacy, with an ORR of 46% and acceptable safety profile. There are also ongoing trials investigating dual LAG3 and PD-1 blockade in HCC. RELATIVITY-073 is a randomised phase II study investigating relatinib (anti-LAG3) and nivolumab *vs.* nivolumab monotherapy in patients with advanced HCC who have progressed on first-line TKI therapy and are immunotherapy-naive (NCT04567615). Unlike many other ICI studies, this trial aims to enrich for those patients most likely to benefit from this combination, with LAG3 expression mandatory for inclusion. Additionally, RELATIVITY-106 (phase I/II) will evaluate the combination of nivolumab, relatinib and bevacizumab compared to nivolumab and bevacizumab alone in the first-line setting (NCT05337137). Finally, use of anti-TIGIT-directed therapy is also being explored in advanced HCC. The three-drug combination of ociperlimab (anti-TIGIT), tislelizumab (anti-PD1) and BAT 1706 (bevacizumab biosimilar) did not improve ORR compared to tislelizumab and BAT1706 alone in a Chinese patient population, although survival data is immature.^105^ In contrast to this, the MORPHEUS-liver study (phase Ib/II) demonstrated a promising ORR of 43.5% when investigating the anti-TIGIT therapy tiragolumab in combination with atezolizumab and bevacizumab,^106^ and this combination has been taken forward into the phase III IMbrave 152 study which commenced recruitment earlier this year (NCT05904886).

### Bispecific antibodies

Unlike monoclonal antibodies, bispecifics are engineered to allow precise binding to two antigens or epitopes, either on the same or different cell types. Bispecific antibodies targeting two different immune checkpoints on T cells (anti-PD-1/CTLA-4 and anti-PD-1/TIGIT) are currently being evaluated in patients with advanced HCC in a phase II study, combining the potential benefits of two drugs in a single molecule (NCT05775159). Alternatively, bispecifics can act as a bridge between effector T cells and tumour cells in order to improve the specificity and effectiveness of cell killing. Application of this technology in HCC remains in its infancy, but a bispecific antibody for GPC3 and the T cell-specific antigen CD3 has been shown to enhance T-cell activation and tumour cell death in HCC cell lines.^107^

### Adoptive cell therapy

Following the success of cell-based immune therapies in haematological malignancies,[108], [109] there has been increasing interest in applying this technology to solid tumours including HCC. Early phase trials have investigated using both gene-modified (*e.g*. chimeric antigen receptor T [CAR-T] cells and T-cell receptor modified T [TCR-T] cells) and non-gene modified adoptive cell therapy (cytokine induced killer [CIK] cells, NK cells and tumour-infiltrating lymphocytes [TILs]).

#### (i) Non-gene-modified cell therapy

CIK cells are CD3^+^CD56^+^ NK-like T cells expanded from peripheral blood that have potential as an “off the shelf” allogeneic therapy. They have shown efficacy in the adjuvant setting in a Korean phase III trial in which patients with early-stage disease treated with resection or ablation were randomised to multiple infusions of autologous CIKs or standard of care.^110^ Allogeneic NK cells have also been investigated in combination with cryoablation in advanced-stage HCC^111^ and in an ongoing phase II trial in combination with targeted therapy (NCT04162158). In contrast, TILs are polyclonal tumour-targeting T cells which are expanded for use as an autologous therapy and have recently gained FDA approval for advanced melanoma. HCC TILs are phenotypically exhausted with high expression of inhibitory immune checkpoints, such as TIM-3 and LAG3,^112^ suggesting that combination therapy with ICIs may be required to maximise their utility in HCC. However, there is a scarcity of clinical trials in this area, with only two studies investigating autologous TILs as an adjuvant therapy after tumour resection.[113], [114] These demonstrated an acceptable toxicity profile, but no further studies of TIL therapy are currently in progress in HCC.

#### (ii) Gene-modified

Gene engineering approaches to cell therapy aim to modify immune cells with synthetic receptors in order to enhance recognition of tumour-specific antigens. CAR-T cells are engineered with synthetic cell surface receptors to enable tumour-specific cell killing in an MHC-independent manner. A growing number of clinical trials are demonstrating the value of CAR-T cells in solid tumours, and several promising targets for CAR-T therapy have been identified in HCC, including GPC3, AFP, NKG2DL (NK group 2 member D ligand), MUC1, CD147, HBV surface protein and c-MET.[115], [116], [117], [118], [119], [120] Currently, most CAR-T cell therapies for HCC are directed at GPC3, due to the favourable combination of high expression in HCC with limited expression in other tissues, including normal and cirrhotic liver. There are multiple ongoing phase I/II trials targeting GPC3 (Table 2) and two sequential phase I studies investigating autologous CAR-GPC3 T cells in advanced HCC have been reported to date.^117^ Although response rates were disappointing with 1/13 achieving a partial response, 2/13 maintaining stable disease and 8/13 progressing on treatment, the toxicity profile was broadly in keeping with published data for CAR-T therapy, with 9/13 (69%) patients experiencing any grade cytokine release syndrome and one death due to cytokine release syndrome (Grade 5). Optimised approaches using armoured CAR-T cell designs, combination therapy with TKIs, ICIs and radiotherapy, and intrahepatic targeted delivery are currently under investigation, with the aim of boosting efficacy. An alternative cell therapy approach in HCC is the use of TCR-T cells, where engineered TCRs are designed to recognise intracellular tumour antigens on HLA class I and II molecules. The advantage of this technique is the additional ability to target intracellular antigens like AFP, which is processed and presented on HLA, although this comes at the cost of limiting therapy to the most frequently shared HLA types. To date, early phase trials of TCR-T in HCC have mostly been directed at AFP or viral associated antigens (predominantly HBV; Table 3). In a phase I study with eight patients, HBV-specific TCR-expressing autologous T cells have demonstrated acceptable tolerability in patients with advanced HBV-related HCC not suitable for liver transplantation, with one patient achieving a durable partial response of 27.7 months.^121^ One concern with using HBV antigens as a target for TCR-T cell therapy is the potential for inducing liver damage due to the expression of viral antigens on non-malignant liver tissue. Potential strategies to circumvent this have utilised mRNA HBV-TCR-directed T cells that are functionally short lived due to the short half-life of mRNA.^122^ Finally, the specificity and high expression of AFP in HCC has been exploited in the development of AFP-directed autologous SPEAR T cells, which have been tested in HLA-A∗02-selected patients with AFP-overexpressing HCC in the phase I setting. Full results are pending, but the initial safety profile appeared favourable, with preliminary evidence of antitumour activity.^123^

**Table 2:** Ongoing trials of CAR T cell therapies.

NCT number	Phase	Target (co-stimulator)	Planned enrolment	Patient population	Sponsor	Region/country	Preconditioning	Primary outcome	Status
**GCP3 based**									
3884751	I	GPC3	15	Advanced HCC	CARsgen Therapeutics Co., Ltd.	China		Safety and tolerability	Completed
3980288	I	GPC3	36	Advanced HCC	Zhejiang Universit	China	Flu + Cyclo	Safety and tolerability	Completed
4121273	I	GPC3	14	Advanced HCC	Baylor College of Medicine	USA	Flu + Cyclo	DLT	Unknown
2959151	I/II	GPC3	20	Advanced HCC	Shanghai GeneChem Co., Ltd	China		Adverse events	Unknown
3146234	I	GPC3	20	Advanced HCC	RenJi Hospital	China	Flu + Cyclo	Safety and tolerability	Completed
5652920	lb/ll	GPC3	105	Advanced HCC	OriCell Therapeutics Co., Ltd.	China		MTD	Recruiting
2715362	l/ll	GPC3 (4-1BB)	30	Advanced HCC	Shanghai GeneChem Co., Ltd	China	Cylco	Safety and tolerability	Unknown
5003895	l	GPC3	38	Advanced HCC	National Cancer Institute (NCI)	USA	Flu + Cyclo	Safety and feasibility	Recruiting
5783570	l	GPC3	12	Advanced HCC	Eutilex	Korea		Adverse events	Recruiting
5103631	l	GPC3 (IL-15)	27	Advanced HCC	Baylor College of Medicine	USA	Flu + Cyclo	DLT	Recruiting
3302403	l	GPC3	48	Advanced HCC	Kang YU	China	Flu + Cyclo	Safety and tolerability	Unknown
5070156	l	GPC3	3	Advanced HCC	Tongji University	China		Adverse events	Not recruiting
6084884	l/ll	GPC3	84	Advanced HCC	AstraZeneca	Korea and USA	Flu + Cyclo	Safety and tolerability	Recruiting
6198296	l	GCP3 (IL-15 and IL-21)	21	Multiple inc HCC	Baylor College of Medicine	USA	Flu + Cyclo	DLT	Not recruiting
5620706	l	GPC3	20	Advanced HCC	Shenzhen University General Hospital	China		Adverse events	Recruiting
5120271	l/ll	GPC3	110	Multiple inc HCC	Sotio Biotech Inc.	USA	Flu + Cyclo	Safety and tolerability	Recruiting
3198546	l	GPC3+/-TGFβ (IL-7)	30	Advanced HCC	Second Affiliated Hospital of Guangzhou Medical University	China		DLT	Recruiting
5155189	l	GPC3	44	Advanced HCC	Zhejiang University	China		Adverse events	Recruiting
4951141	l	GPC3	10	Advanced HCC	Beijing Immunochina Medical Science & Technology Co., Ltd.	China		Adverse events	Unknown
2395250	l	GPC3	10	Advanced HCC	RenJi Hospital	China		Adverse events	Completed
3084380	l/ll	GPC3	20	Advanced HCC	Xinqiao Hospital of Chongqing	China	Flu + Cyclo	Safety	Unknown
6144385	l	GPC3	20	Advanced HCC	Shanghai Ming Ju Biotechnology Co., Ltd.	China	Flu + Cyclo	Safety	Recruiting
5926726		GPC3	12	Advanced HCC	RenJi Hospital	China	Flu + Cyclo	DLT and adverse events	Recruiting
**Non-GCP3 targets**									
3672305	l	c-Met/PD-L1	50	Advanced HCC	The Second Hospital of Nanjing Medical University	China	Flu + Cyclo	Efficacy	Unknown
5323201	l/ll	B7H3	15	Advanced HCC	The Affiliated Hospital of Xuzhou Medical University	China	Flu + Cyclo	Safety and response rate	Recruiting
3013712	l/ll	EPCAM	60	Multiple inc HCC	First Affiliated Hospital of Chengdu Medical College	China		Toxicity	Unknown
3993743	l	CD147	34	Advanced HCC	Xijing Hospital	China		Adverse events	Unknown
5028933	l	EPCAM	48	Multiple inc HCC	Zhejiang University	China	Flu + Cyclo	PK and adverse events	Recruiting
5131763	l	NKG2DL (4-1BB)	3	Multiple inc HCC	Fudan University	China		Adverse events	Unknown
4550663	l	NKG2DL	10	Multiple inc HCC	The Affiliated Nanjing Drum Tower Hospital of Nanjing University	China		MTD and adverse events	Unknown
2587689	l	MUC1	20	Multiple inc HCC	PersonGen BioTherapeutics (Suzhou) Co., Ltd.	China		Adverse events	Unknown
3941626	l/ll	EGFRvIII/DR5	50	Multiple inc HCC	Shenzhen BinDeBio Ltd.	China	Flu + Cyclo	Adverse events	Unknown
3638206	l/ll	EGFRvIII/DR5/C-met	73	Multiple inc HCC	Shenzhen BinDeBio Ltd.	China	Flu + Cyclo	Adverse events	Unknown

Cyclo, cyclophosphamide; DLT, dose limiting toxicity; Flu, fludarabine; GPC3, glypican 3; MTD, maximum tolerated dose; NR, not reported.

**Table 3:** Ongoing genetically engineered TCR T-cell therapies in progress.

NCT number	Phase	Target	Planned enrolment	Patient population	HLA class (if stated)	Sponsor	Region/country	Primary outcome	Status
4745403	l	HBV Ag	10	HBV Ag + advanced HCC	HLA-A∗02:01 or HLA-A∗24:02	Lion TCR Pte. Ltd.	Singapore	Safety	Recruiting
3899415	l	HBV Ag	10	HBV Ag + advanced HCC		Beijing 302 Hospital	China	Safety	Recruiting
4677088	l	HBV Ag	7	HBV Ag+ HCC post- transplant		Xiaoshun He	China	Safety	Unknown
2686372	l	HBV Ag	13	HBV Ag+ HCC post -transplant		Lion TCR Pte. Ltd	China	Adverse events	Completed
5339321	l	HBV Ag	36	HBV Ag + advanced HCC	HLA-A∗02:01	Peking Union Medical College Hospital	China	Adverse events	Unknown
5195294	l/ll	HBV Ag	55	HBV Ag + advanced HCC		Lion TCR Pte. Ltd	NR	Adverse events	Not recruiting
5417932	l/lla	HBV Ag	46	HBV Ag + advanced HCC	HLA-A∗02:01	SCG Cell Therapy Pte. Ltd	Hong Kong, Singapore, USA	Safety and response	Recruiting
3971747	l	AFP	9	HCC serum AFP >200 ng/ml	HLA-A∗02:01	Cellular Biomedicine Group Ltd.	China	Adverse events	Unknown
4368182	l	AFP	3	HCC serum AFP >200 ng/ml	HLA-A∗02:01	Zhejiang University	China	Safety	Unknown
3132792	l	AFP	30	HCC serum AFP ≥100 ng/ml	HLA-A∗02:01	Adaptimmune	EU and USA	DLT and adverse events	Completed

AFP, alpha-fetoprotein; HBV, hepatitis B virus; HCC, hepatocellular carcinoma.

## Conclusion

Immune checkpoint inhibition has transformed the management of advanced HCC and emerging data suggest a possible role for its use in earlier disease stages. These data will require robust evaluation and have implications for subsequent treatment in the advanced setting, including sequencing of therapy and the role of continuing ICI beyond disease progression for patients with clinical benefit (as currently being evaluated in the phase III IMBRAVE 251 study [NCT04770896]). Despite these advances, there is currently insufficient evidence to guide selection of those patients most likely to benefit from ICIs and questions remain as to whether new ICI-based combinations will be able to overcome resistance to atezolizumab/bevacizumab treatment in the first-line setting. Ongoing studies evaluating novel combinations and alternative immunotherapeutic strategies are looking to answer these questions and improve the survival benefit already demonstrated in advanced disease. Recruitment to clinical trials with embedded translational research will be key in building upon the success seen to date and improving patient outcomes.

## Abbreviations

ABRS, atezolizumab plus bevacizumab response signature; AFP, alpha-fetoprotein; CAR-T, chimeric antigen receptor-T; CIK, cytokine-induced killer; CTLA-4, cytotoxic T-lymphocyte associated protein 4; CXCL, chemokine C-X-C motif ligand; DOR, duration of response; GPC3, glypican 3; HR, hazard ratio; ICI, immune checkpoint inhibitor; IFN, interferon; IL-, interleukin-; LAG3, lymphocyte-activating 3; MDSCs, myeloid-derived suppressor cells; NK, natural killer; ORR, objective response rate; OS, overall survival; PD-1, programmed death 1; PD-L1, programmed death ligand 1; PFS, progression-free survival; TAMs, tumour-associated macrophages; TCR-T, T-cell receptor modified-T; TGFβ, transforming growth factor-β; TIL(s), tumour-infiltrating lymphocyte(s); TIGIT, T cell immunoreceptor with Ig and ITIM domains; TKI(s), tyrosine kinase inhibitor(s); TMB, tumour mutational burden; TME, tumour microenvironment; Tregs, regulatory T cells.

## Financial support

The authors did not receive any financial support to produce this manuscript. GAM is funded by Cancer Research UK HUNTER, Ref. C9380/A26813. TM is funded by National Institute for Health Research (NIHR203950) and NIHR UCH Biomedical Research Facility.

## Conflict of interests

T. Meyer reports Consultancy: Roche, Astra Zeneca, Signant Health, GreyWolf, Guerbet, Geneos, Eisai, Beigene, MSD. Research Funding: MSD, Bayer, Boston Scientific.

Please refer to the accompanying ICMJE disclosure forms for further details.

## Authors’ contributions

1. Concept and design – All. 2. Drafting of manuscript- All. 3. Approval of final manuscript- All.
